# Multiple Coexisting Species and the First Known Case of a Cheater in *Epicephala* (Gracillariidae) Associated with a Species of *Glochidion* (Phyllanthaceae) in Tropical Asia

**DOI:** 10.1093/jisesa/ieaa081

**Published:** 2020-08-25

**Authors:** Zhibo Wang, Xiaofei Yang, Zhenguo Zhang, Fuchen Shi, Houhun Li

**Affiliations:** College of Life Sciences, Nankai University, Tianjin, China

**Keywords:** cheater, coevolution, *Epicephala*, *Glochidion sphaerogynum*, mutualistic

## Abstract

*Glochidion* plants and *Epicephala* moths played different roles and kept the balance in the mutualism. We studied the four coexisting *Epicephala* species on *Glochidion sphaerogynum* in detail and reconstructed the phylogenic tree of 40 Gracillariidae species. The results showed that one of them (*Epicephala impolliniferens*) did not pollinate *G. sphaerogynum*, because of lacking the specialized structure of carrying pollen. These results suggested that *E. impolliniferens* acted as a ‘cheater’ in the system. The phylogenetic analyses suggested that *E. impolliniferens* derived from a pollinating species, and had secondarily gave up the ability to pollinate. This is a typical phenomenon of mutualism reversal. The phenomenon exhibits the co-evolutionary diversification under selection pressures.

Mutualistic relationships between phytophagous insects and their host plants is a well-studied model of co-evolution (Anna and Johan 2001). Among these mutualisms, the fig–fig wasp (Wiebes 1979, Weiblen 2002, Cook and Rasplus 2003) and yucca–yucca moth (Bogler et al. 1995, Huth and Pellmyr 1999, Pellmyr 2003) relationships have become classic models, in which the insect actively collect pollen and pollinate a specific plant, and the larvae feed on the seeds of corresponding hosts. In these models, pollinators and host plants have high species specificity, meaning that the host plant generally has the exclusive pollinator. However, it also was reported that this exclusive relationship had been broken, especially in the fig–fig wasp. Non-pollinating cheaters have also been found in these mutualisms (Addicott 1996, Segraves et al. 2005). In fact, those cheaters turned the symbiotic relation with pollinators. In mutualistic systems, pollinator can pollinate the host as cost to exchange the benefit that their larvae feed seeds of host, while cheaters also acquired the same benefit without providing any pollination service (Kevin and Hanna 2006). The founding of cheaters is very important in the study of the co-evolution of mutualism (Addicott 1996). In the mutualism between *Glochidion* plants and *Epicephala* moths, female flowers have a specialized united style that cannot be pollinated by generalist insect pollinators. While female moths can actively pollinate female host using their ciliated proboscis and lay eggs in the ovaries using their specialized ovipositor, and their larvae then feed on part of seeds in the fruit (Kato et al. 2003, Kawakita and Kato 2004, Kawakita 2010). The plants of host include species in the genera *Glochidion*, *Breynia*, and *Phyllanthus* (Kato et al. 2003, Kawakita 2010, Zhang et al. 2012). Although *Epicephala* moths and their hosts tend to present one-to-one specificity, there are some exceptions, as evidenced for other systems. For example, two *Epicephala* species copollinated *Glochidion lanceolatum* plants and another two *Epicephala* species shared *G. obovatum* and *G. rubrum* plants (Kawakita and Kato 2006), two *Epicephala* species shared plants of two *Breynia* species (Zhang et al. 2012), and there also have reported that one *Epicephala* species has spread across 12 host species in Southeastern Polynesia (Hembry et al. 2013). In addition, the seed-parasitic *Epicephala* species that did not pollinate the flowers in *Flueggea suffruticosa* and three herbaceous *Phyllanthus* species had been reported (Kawakita and Kato 2009). These examples mentioned earlier showed the co-evolutionary diversification in leafflower–leafflower moth association.

Here we describe a cheater in a more complex mutualistic system in which four different *Epicephala* species co-occurr on *G. sphaerogynum* (Müller Argoviensis) Kurz. The cheater had lost the ability of pollination. This study showed us a better understanding on how mutualisms can evolve into antagonisms, including where cheaters come from, how cheating evolves, and the natural history of cheaters. Furthermore, *E. impolliniferens* may become the pattern species of cheater used in co-evolution of mutualism in future.

## Materials and Methods

We observed the phenology and morphology of 75 *Glochidion sphaerogynum* trees (Li and Gilbert 2008) in the Yinggeling Nature Reserves in Hainan, China (19°02′21″N, 109°34′04″E, 450 m). The behaviors of adult *Epicephala* also were studied on *G. sphaerogynum* by the field observations. Most of observations were made during the flowering and fruiting seasons of the plants to examine the behavior and different roles of the *Epicephala*.

Those observations included four time periods: 7:00–11:30, 12:00–17:30, 18:00–23:00, and 23:30–6:30. The total observation time was >1,000 h. The behavior of *Epicephala* was photographed by using a digital camera (Cannon G11). It is almost impossible to distinguish different *Epicephala* species by external characteristics. We recorded the behavior of moths during observation and then captured them using a rearing tube (5.5 cm in length, 1.5 cm in diameter), preserved them in ethanol (absolute) or dried them and brought them back to the laboratory for identification by dissecting the genitalia. In this way, we associated each *Epicephala* species with its behavior. For the flower-visiting moths, we checked whether they carried pollen on their proboscises by microscope (Olympus SZ11). The structures of the female proboscises of *Epicephala* were photographed by microscope (Leica DM750 plus Leica Application Suite 4.2 software). The proboscises with pollen were then photographed by SEM (Quanta 200).

Mature fruits were collected from *G. sphaerogynum*, and the larvae existing in the fruits were reared in order to evaluate the quantity of different species. The mature fruits were collected from *G. sphaerogynum* and stored in rearing containers (11 cm in height, 8 cm in diameter; ≤100 fruits per container). The containers were ventilated and cleaned every 12 h. The larvae were transferred into a new rearing tube (5.5 cm in length, 1.5 cm in diameter) to pupate if they crawled out of the fruit when the containers were being cleaned. This method allowed us to also observe whether there were other insects parasitizing the larvae. Adult moths were divided into two groups. One group was stored in ethanol (absolute) for molecular analysis when the moths were alive; another group was made into dry specimens for taxonomic research (Landry and Landry 1994).

The genitalia of the moths were dissected to identify the species First, the abdomen of each dried *Epicephala* specimen was removed with tweezers and placed in 10% KOH and heated for 10 min to dissolve the muscular tissue. The abdomen was then transferred to distilled water to remove residual tissue and stained in eosin for 24 h. Genitalia were separated from the abdomen and placed in ethanol (absolute) to dehydrate. After dehydration, the genitalia were flattened and fixed in dimethyl benzene for 1 min; mounted in Canada balsam and dried in an oven (Li and Zheng 1996).

The pollen grains were examined on proboscis of female moths by microscopy. Vouchers of plant and *Epicephala* moths have been deposited in the insect collection, College of Life Sciences, Nankai University. The quantity of the four *Epicephala* species were compared by χ ^2^ test implemented in R package.

DNA of the four *Epicephala* species was extracted from the head, thorax, and legs using the saturation sodium chloride method (Zimmermann et al. 2000), and the gene fragments of mitochondrial cytochrome *c* oxidase subunit I (*CO1*), nuclear arginine kinase (*ArgK*), elongation factor-1 (*Ef1-alfa*), were PCR amplified using specific primers (Kawakita and Kato 2004, Ye et al. 2013) described previously (Supp Table 1 [online only]). The newly obtained sequences have been deposited in the GenBank database and the gene sequences of other 36 moth species were acquired from GenBank (Supp Table 2 [online only]). Phylogenetic relationships were established based on sequences of *CO1* (444 bp), *ArgK* (591 bp), and *Ef1-alfa* (469 bp). *Melanocercops ficuvorella* and *Stomphastis labyrinthica* were chosen as outgroups (Kawakita and Kato 2009). Gene sequences were aligned using CLUSTAL 1.83. The optimal model was selected by JModelTest 0.1.1. Phylogenetic analyses were conducted using two different methods. Bayes and maximum-likelihood trees were built by MrBayes v32 and PAUP 4.0b10, respectively. Bootstrap analysis was performed with 1,000 replicates for the maximum-likelihood tree.

## Results

### Coexisting on the host of *G. sphaerogynum*

In total, 705 *Epicephala* adults were reared from larvae founding in fruits of *G. sphaerogynum* population in the Yinggeling Nature Reserves. Based on the taxonomic study by dissecting and comparing the genitalia, those adults were identified as four different *Epicephala* species, *E. domina*, *E. impolliniferens*, *E. angustisaccula*, and *E. camurella* (Li et al. 2015). All larvae feed on seed of *G. sphaerogynum*. *Epicephala domina* was the most prevalent species, accounting for 88.51%; *E. impolliniferens* was the second abundant species, accounting for 10.78%; four individuals (0.57%) were identified as *E. angustisaccula*; and one (0.14%) was found to be *E. camurella* (Table 1). The quantity of the four *Epicephala* species were significantly different on *G. sphaerogynum* (x^2^ = 1,537, *P* < 2.2e-16).

**Table 1.** The quantity of four *Epicephala* species existing on *G. Sphaerogynum*

Sex	*E. angustisaccula*	*E*. *impolliniferens*	*E*. *domina*	*E*. *camurella*
Female	0	39	381	0
Male	4	37	243	1
Total	4	76	624	1

### Behaviors of *Epicephala*

**Fig. 1.**
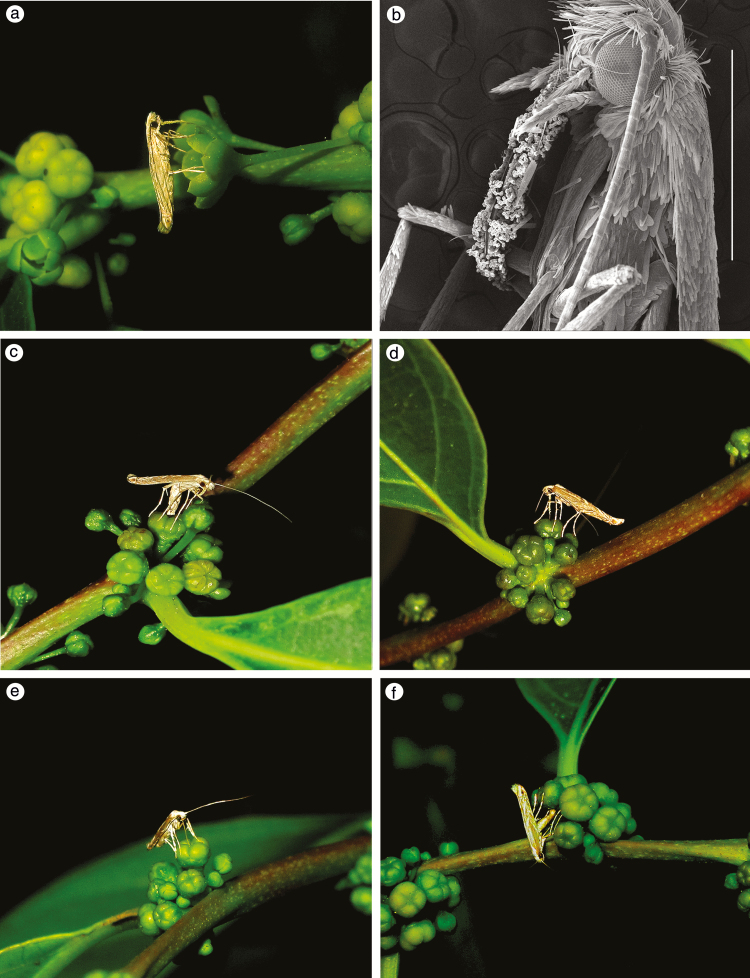
Female *Epicephala* species ovipositing on and pollinating *G. sphaerogynum* flowers. (a) *E. domina* collecting pollen on a male flower. (b) SEM of pollen sticking on an *E. domina* proboscis, scale bar = 1.0 mm. (c) *E. domina* ovipositing by inserting its ovipositor in depression on top of the female flower. (d) *E. domina* pollinating a female flower after ovipositing. (e) Checking behavior of *E. impolliniferens*. (f) *E. impolliniferens* ovipositing by inserting its ovipositor at the base of a style.

Adult moths became active at dusk. Usually, when female moth searching flowers, the antennae were always shaking in a repeating cycle. As a female *E. domina* moth, it chose a male flower and rubbed its proboscis against stamens to collect pollen (Fig. 1a and b). The male flowers of *G. sphaerogynum* grow at the base of branchlets, whereas the female flowers become more abundant towards the branch apex. The female *E. domina* moth showed a checking behavior before oviposition by introducing their proboscis into depression on top of the female flower (number of observed female *E. domina* checking female flowers = 345). The checking behavior repeated among different female flowers till a suitable one was chosen. And then it bent its abdomen and inserted ovipositor into depression on top of the female flower (Fig. 1c). The oviposition lasted 60–90 s (*n* = 143). At the end of oviposition, female *E. domina* curled its proboscis to move pollen from the base to the tip of proboscis (*n* = 143). It then removed ovipositor and inserted proboscis into depression on the top of the female flower and pollinate the female flower (Fig. 1d). It took 30–40 s (*n* = 143) for a female *E. domina* to complete this pollination process. We found pollen grains on proboscises for those *E. domina* moths with pollinating behavior by Olympus Z11 (*n* = 40).

In contrast, female *E. impolliniferens* were not observed collecting pollen on male flowers and pollinating female flowers, although these moths oviposited in female flowers. Female *E. impolliniferens* showed similar female flower checking behavior (Fig. 1e) before oviposition, which was sustained short time about 1–2 s and without the behavior of shaking the head. Once *E. impolliniferens* selected a female flower, it inserted its ovipositor into the base of the style (Fig. 1f) for 60–120 s (*n* = 21). However, we did not observe female *E. angustisaccula* and *E. camurella* oviposition or pollinating on *G. sphaerogynum*.

In addition, we examined the pollen grains on proboscises for female moths by microscopy. Pollen grains were observed on proboscises for those *E. domina* moths with pollinating behavior (*n* = 40), while no pollen grains were observed on the proboscises of female *E. impolliniferens* which displayed such ovipositing behavior (*n* = 14). More importantly, There were cilia on proboscis of *E. domina* but no cilia on the proboscis of *E. impolliniferens* (Li et al. 2015).

### Phylogenetic Analyses

**Fig. 2.**
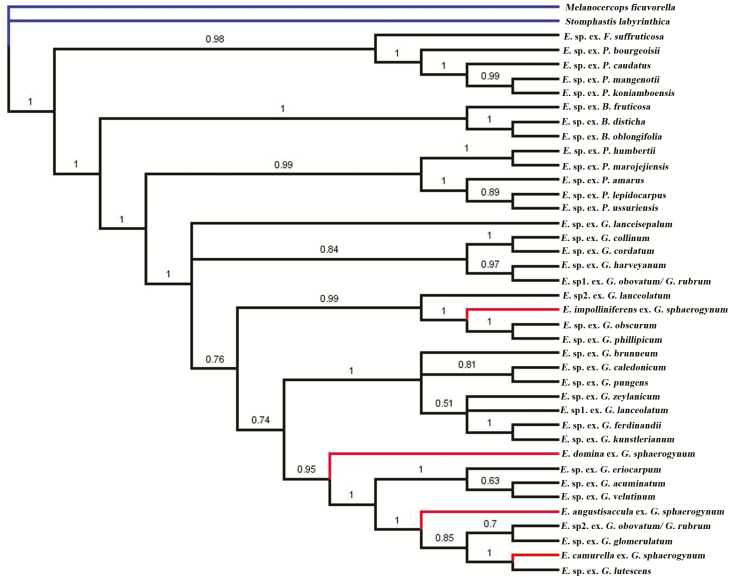
Bayesian tree of 40 Gracillariidae moth species. Analyses consisted of running four simultaneous chains for 1 × 106 generations, sampling trees every 100 generations for a total of 10,001 trees. We discarded 2,501 trees as the burn-in region. Branch labels represent posterior probabilities. Blue branches refer to outgroups, and red branches refer to those four species focused in this study.

We reconstructed the phylogeny of 40 Gracillariidae species. The aligned gene sequences were composed of a 1,504 bp region including *CO1* (444 bp), *ArgK* (591 bp), and *Ef1-alfa* (469 bp). The TIM1ef +I+G model was selected as the optimal model based on the Akaike information criterion. Bayesian and maximum-likelihood trees were built based on the 40 aligned gene sequences (Fig. 2). Bayesian phylogenetic analysis showed that *E. domina*, *E. impolliniferens*, *E. camurella*, and *E. angustisaccula* nested in the clade of members that pollinate *Glochidion*, and the relationships of the four *Epicephala* species were paraphyletic. *Epicephala domina*, *E. camurella*, and *E. angustisaccula* fell into the same clade, which suggests that the three *Epicephala* species have an intimate phylogenetic relationship. In contrast, *E. impolliniferens* had a more distant relationship with the other three *Epicephala* species. The maximum-likelihood analyses showed similar results with the Bayesian tree, but with lower supported resolution (Supp Fig. 1 [online only]).

## Discussion

### Behavior of Adult *Epicephala*

When searching flowers, the antennae of female moth were always shaking in a repeating cycle. This movement likely engages the large number of odor receptors on the antennae, allowing the moths to locate flowers (Okamoto et al. 2007, Svensson et al. 2010, Kawahara et al. 2013). We found both *E. domina* and *E. impolliniferens* showed the behavior of checking female flower before oviposition, the checking behavior can be distinguished with pollinating behavior by the difference of duration. Those moths maybe can identify if the female flower have been oviposited by the checking behavior and then make the choice of oviposition. The checking behavior may avoid repetitive ovipositing in a female flower for *E. domina* (Wang and Li 2015), while help selecting a pollinated female flower for *E. impolliniferens* based on the fact no exceeding three larvae in a fruit of *G. sphaerogynum* which was common phenomenon in *Phyllanthus microcarpus* (Yang and Li 2015). Alternatively, there was obvious difference in oviposition sites between *E. impolliniferens* and *E. domina*. That is the best way to distinguish the two species in the field. The difference of oviposition sites may result in shape of ovipositors. The valvule was arrowhead shaped in *E. impolliniferens* but spade shaped in *E. domina* (Li et al. 2015). The valvule of arrowhead shaped is common in the genus of *Epicephala* (Li et al. 2015, Yang and Li 2015). That was sharper than spade shaped one and may allow *E. impolliniferens* to perforate the ovary wall. The difference in ovipositors of the two *Epicephala* species may result from the difference in oviposition site between *E. impolliniferens* and *E. domina*.

### A Cheater in *Glochidion*–*Epicephala* Mutualism

*Epicephala domina* is the dominant species and they pollinated *G. sphaerogynum* (Table 1; Wang and Li 2015). And *E. impolliniferens* is the second abundant species. As it reported that the cilia of proboscises possessed the function of carrying pollen (Kato et al. 2003). But no cilia were found on female *E. impolliniferens* (Li et al. 2015), which was consistent with the fact that no pollen grains were found on the proboscises of female *E. impolliniferens*. Most important, *E. impolliniferens* did not show pollinating behavior. The evidence above suggests that *E. impolliniferens* is a cheater which is known as only consuming but no offering in the mutualism, then it is not mutualistic partner. This is the first time a cheater species reported in the *Glochidion*–*Epicephala* mutualistic co-evolutionary system.

### The Importance of Specialized Structure

Generally, the specialized structures serve exclusive function. In yucca moth, the unique structure of tentacular mouthparts has the function to carry pollen, and some yucca moths gave up pollination by oviposition later in flower development and as a consequence lack tentacle (Addicott 1996, Pellmyr and Krenn 2002). It has never been reported that yucca moth can pollinate without tentacles (Pellmyr 2003). In fig–fig wasp, pollinator also possess the unique features associated with pollen transport such as corbiculae on the forecoxae, pockets on the mesothorax or grooves between the abdominal segments (Ramirez 1978). It was reported the female moths have evolved to actively collect and transport pollen between flowers using specialized proboscides equipped with numerous sensilla (Kawakita and Kato 2006, 2009). However, there also had reported that two *Epicephala* species that lost cilia on their proboscis could still actively pollinate their host (Kawakita et al. 2015), which is contradictory to previous result. The specialized structure and the exclusive function should be consistent. Thus, *Epicephala* species which lost cilia on their proboscis should lose the actively pollinating ability.

### Origin of the Cheater

Cheaters have been observed in other typical mutualistic co-evolutionary systems such as the fig–fig wasp and yucca–yucca moth (Compton et al. 1991, Pellmyr and Thompson 1992, Althoff et al. 2004, Peng et al. 2005, Al-Beidh et al. 2012, Chen et al. 2013). In those systems, cheaters, pollinators, and hosts maintain a close interaction. Cheaters may arise from various evolutionary pathways (Pellmyr et al. 1996). Yucca moths became active pollinators after the colonization of yuccas and before the divergence of two pollinator genera (Pellmyr et al. 1996). That suggests two possible pathways for the origin of the cheater species: they were retained from the original nonpollinating lineage or they evolved from the pollinators (Pellmyr et al. 1996, Pellmyr and Leebens-Mack 2000, Yu 2001, Machado et al. 2005). Our phylogenetic analyses showed that *E. impolliniferens* nested in the clade of species that pollinate *Glochidion*, suggesting that *E. impolliniferens* was in the pollinating cladogram and nonpollinating species, but it lost pollinating habit and cilia on the proboscis in the course of evolution. So, this is a typical phenomenon of mutualism reversal (Pellmyr and Leebens-Mack 2000, Kawakita et al. 2015).

We proposed a hypothesis about the origin of *E. impolliniferens* that the cheater *E. impolliniferens* derived from the pollinator *E. domina*. In this case, the relationship between them should be monophyletic, but we found that they were paraphyletic instead. Therefore, we concluded that *E. impolliniferens* did not derive from the *E. domina* based on the potential relationship in the cladogram. On other hand, host shift may explain of how the cheater and pollinator coexist on the same host, and the host shift also may become a new pathway for evolution of species (Kawakita and Kato 2009, Yang et al. 2012, Hembry et al. 2013).

## Conclusions

Four *Epicephala* species coexist on *G. sphaerogynum* in what appears to be a complicated co-evolutionary relationship. *E. domina* was the dominant species and could actively pollinate *G. sphaerogynum*. As a cheater in the system, *E. impolliniferens* originated from a pollinating *Epicephala* species but gave up pollinating ability and habit in the course of evolution. The phenomenon of cheater is a typical example of mutualism reversal in evolution. In our present study, we did not find what evolutionary drive made the *E. impolliniferens* turning from pollinator to cheater; however, our findings broke away a hypothesis of homologous evolution between pollinator and cheater. That may bring us more understanding about the diversification of origin for cheater in a mutualism.

## Supplementary Material

ieaa081_suppl_Supplementary_File
