# Supplementary material for: Multiple Coexisting Species and the First Known Case of a Cheater in Epicephala (Gracillariidae) Associated with a Species of Glochidion (Phyllanthaceae) in Tropical Asia
Source: J Insect Sci. 2020 Aug 25;20(4):23. doi: 10.1093/jisesa/ieaa081 (PMC7447135; doi:10.1093/jisesa/ieaa081)
Supplement: ieaa081_suppl_Supplementary_File [file ieaa081_suppl_supplementary_file.docx]

**Supplementary file
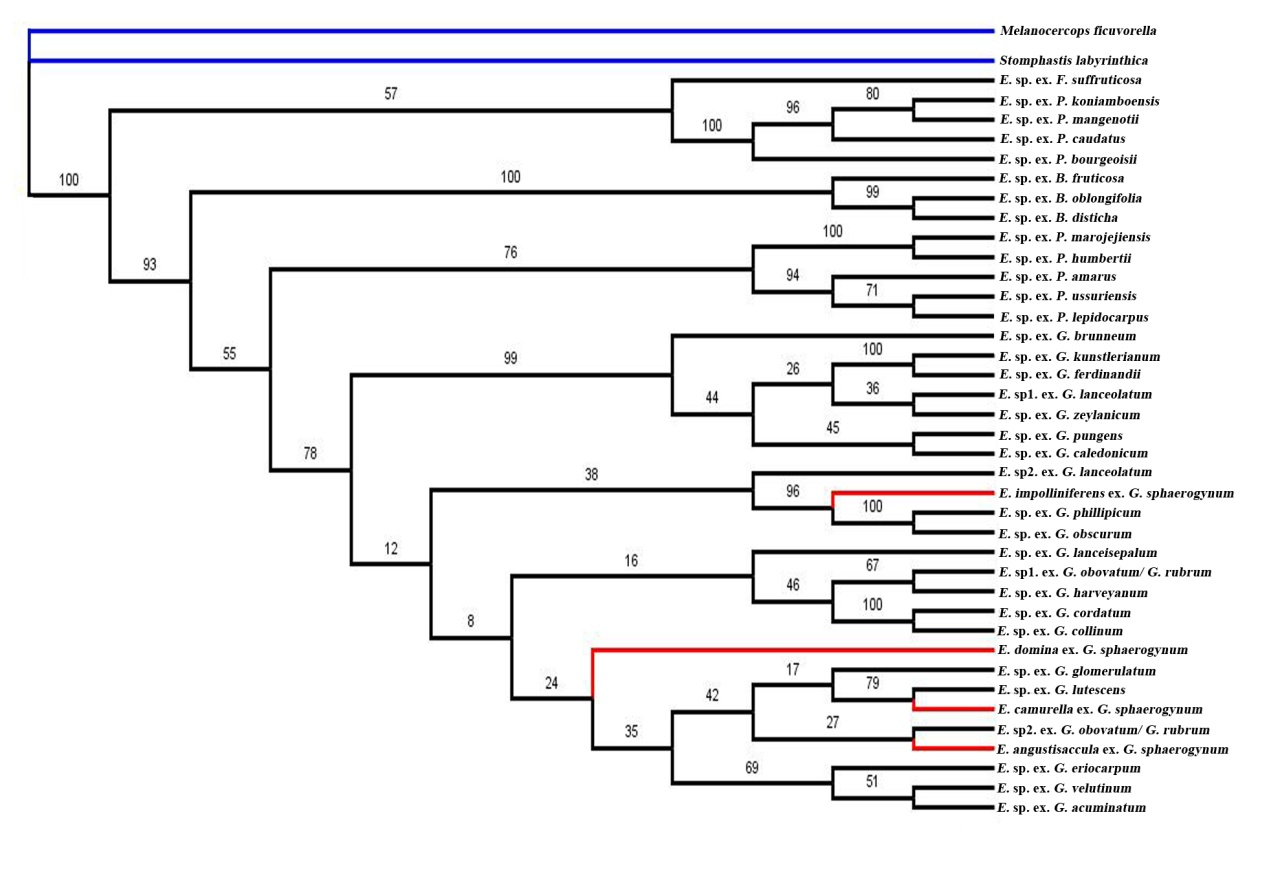
**

**Figure S1:** Maximum likelihood tree of 40 Gracillariidae moth species. Branch labels represent bootstrap support values. Blue branches refer to outgroup, and red branches refer to those four species focused in this study.

**Table S1:** A list of primers used in the present study.

| **Locus** | **Primer name** | **Sequence (5’- 3’)** | **Reference** |
| --- | --- | --- | --- |
| *CO1* | F2 Forward | AYTCWACMAATCATAAAGATATTGG | Ye et al. 2013 |
|  | F1 Reverse | TAAACTTCTGGATGWCCAAAAAAT | Ye et al. 2013 |
| *ArgK* | ArgK-F4 | ATTTAGACTCTGGTGTTGG | Kawakita et al. 2004 |
|  | ArgK-R4 | ATGCCGTCGTACATCTCCTT | Kawakita et al. 2004 |
| *Ef1-alfa* | ef1af2 | CCCATTTCKGGCTGGCAYGGAGA | Kawakita et al. 2004 |
|  | ef1ar2 | GATTTACCRGWACGACGRTC | Kawakita et al. 2004 |

**Table S2:** GenBank accession numbers of 40 Gracillariidae moth species including *CO1*, *ArgK* and *Ef1-alfa* sequences. *Sequences submitted from this study; #Outgroup sequences.

| **No.** | **Species** | **Genbank accession number** | | |
| --- | --- | --- | --- | --- |
|  |  | ***CO1*** | ***ArgK*** | ***Ef1-alfa*** |
| 1 | *E.* sp. ex. *G. caledonicum* | AY525720.1 | AY525740.1 | AY538753.1 |
| 2 | *E.* sp. ex. *G. collinum* | AY525721.1 | AY525741.1 | AY538754.1 |
| 3 | *E.* sp. ex. *G. cordatum* | AY525723.1 | AY525743.1 | AY538756.1 |
| 4 | *E.* sp. ex. *G. ferdinandii* | AY525724.1 | AY525744.1 | AY538757.1 |
| 5 | *E.* sp. ex. *G. harveyanum* | AY525725.1 | AY525745.1 | AY538758.1 |
| 6 | *E.* sp. ex. *G. lanceisepalum* | AY525726.1 | AY525746.1 | AY538759.1 |
| 7 | *E.* sp1. ex. *G. lanceolatum* | DQ298995.1 | DQ452199.1 | DQ452267.1 |
| 8 | *E.* sp2. ex. *G. lanceolatum* | DQ298996.1 | DQ452205.1 | DQ452271.1 |
| 9 | *E.* sp. ex. *G. acuminatum* | DQ298956.1 | DQ452157.1 | DQ452241.1 |
| 10 | *E.* sp1. ex. *G. obovatum/ G. rubrum* | DQ299017.1 | DQ298910.1 | DQ299116.1 |
| 11 | *E.* sp2. ex. *G. obovatum/ G. rubrum* | DQ299004.1 | DQ452222.1 | DQ452287.1 |
| 12 | *E.* sp. ex. *G. phillipicum* | AY221980.1 | AY525749.1 | AY538762.1 |
| 13 | *E.* sp. ex. *G. pungens* | AY525730.1 | AY525750.1 | AY538763.1 |
| 14 | *E.* sp. ex. *G. velutinum* | AY525734.1 | AY525754.1 | AY538767.1 |
| 15 | *E.* sp. ex. *G. zeylanicum* | DQ299036.1 | DQ452233.1 | DQ452289.1 |
| 16 | *E.* sp. ex. *B. fruticosa* | FJ235379.1 | FJ235403.1 | FJ235502.1 |
| 17 | *E.* sp. ex. *B. disticha* | FJ235378.1 | FJ235402.1 | FJ235501.1 |
| 18 | *E.* sp. ex. *B. oblongifolia* | FJ235381.1 | FJ235405.1 | FJ235504.1 |
| 19 | *E.* sp. ex. *P. mangenotii* | AY269398.1 | FJ235393.1 | FJ235492.1 |
| 20 | *E.* sp. ex. *P. koniamboensis* | FJ235374.1 | FJ235394.1 | FJ235493.1 |
| 21 | *E.* sp. ex. *P. caudatus* | FJ235375.1 | FJ235396.1 | FJ235495.1 |
| 22 | *E.* sp. ex. *P. bourgeoisii* | AY269410.1 | FJ235395.1 | FJ235494.1 |
| 23 | *E.* sp. ex. *P. amarus* | FJ235388.1 | FJ235412.1 | FJ235511.1 |
| 24 | *E.* sp. ex. *P. ussuriensis* | FJ235387.1 | FJ235411.1 | FJ235510.1 |
| 25 | *E.* sp. ex. *P. lepidocarpus* | FJ235386.1 | FJ235410.1 | FJ235509.1 |
| 26 | *E.* sp. ex. *P. marojejiensis* | FJ235384.1 | FJ235408.1 | FJ235507.1 |
| 27 | *E.* sp. ex. *P. humbertii* | FJ235385.1 | FJ235409.1 | FJ235508.1 |
| 28 | *E.* sp. ex. *G. glomerulatum* | KC912962.1 | KC912904.1 | KC913018.1 |
| 29 | *E.* sp. ex. *G. kunstlerianum* | KC912966.1 | KC912908.1 | KC913022.1 |
| 30 | *E.* sp. ex. *G. brunneum* | KC912960.1 | KC912902.1 | KC913016.1 |
| 31 | *E.* sp. ex. *G. lutescens* | KC912968.1 | KC912910.1 | KC913024.1 |
| 32 | *E.* sp. ex. *G. obscurum* | KC912971.1 | KC912913.1 | KC913027.1 |
| 33 | *E.* sp. ex. *G. eriocarpum* | KC912959.1 | KC912901.1 | KC913015.1 |
| 34 | *E.* sp. ex. *F. suffruticosa* | FJ235373.1 | FJ235392.1 | FJ235491.1 |
| 35 | *E. domina* ex. *G. sphaerogynum ** | MT857208 | MT857207 | MT890753 |
| 36 | *E. impolliniferens* ex. *G. sphaerogynum ** | MT857206 | MT857205 | MT857204 |
| 37 | *E. camurella* ex. *G. sphaerogynum ** | MT857213 | MT857209 | MT857214 |
| 38 | *E. angustisaccula* ex. *G. sphaerogynum ** | MT857212 | MT857210 | MT857211 |
| 39 | *Melanocercops ficuvorella* # | FJ235391.1 | FJ235415.1 | FJ235514.1 |
| 40 | *Stomphastis labyrinthica* # | FJ235390.1 | FJ235414.1 | FJ235513.1 |

*Sequences submitted from this study; #outgroup sequences.
